# The role of dapoxetine hydrochloride on-demand for the treatment of men with premature ejaculation

**DOI:** 10.1038/srep07269

**Published:** 2014-12-01

**Authors:** Cao De Hong, Liu Liang Ren, Huang Yu, Wei Qiang

**Affiliations:** 1 Department of Urology, West China Hospital, Sichuan University, China

## Abstract

Premature ejaculation (PE) is the most common male sexual dysfunction. Dapoxetine hydrochloride, belonging to a class of drugs known as selective serotonin reuptake inhibitors or, was the first drug originally approved for the on-demand treatment of men with PE. We aimed to compare the intravaginal ejaculatory latency time (IELT), patient-reported global impression of change (PGIC), and adverse effect (AE) incidence associated with the use of dapoxetine (30 mg and 60 mg) versus placebo, and evaluate the differences in administering 60 mg versus 30 mg as on-demand medical oral therapy for the treatment of PE via a literature review and meta-analysis. Relevant randomized controlled trials (RCTs) were identified from PubMed, EMBASE, and Cochrane Central Register of Controlled Trials (Cochrane Library) databases. Ultimately, a total of seven RCTs with 8039 patients were included. Our meta-analysis demonstrated that dapoxetine (in the 30 mg and 60 mg subgroup) resulted in significantly higher IELT, PGIC, and AE incidence relative to the placebo, with higher proportions observed for 60 mg versus 30 mg of dapoxetine administration. The most common AEs were mild and tolerable. We conclude that dapoxetine (particularly the 60 mg dosage) may be considered a safe and effective drug for patients with PE.

Premature ejaculation (PE) is the most common male sexual dysfunction with a prevalence of between 20% and 40%^1^,^2^. Recently, the International Society for Sexual Medicine has proposed the following evidence-based definition: “PE is a male sexual dysfunction characterized by ejaculation that always or nearly always occurs prior to or within about 1 minute of vaginal penetration; inability to delay ejaculation on all or nearly all vaginal penetrations; and negative personal consequences, such as distress, bother, frustration, and/or the avoidance of sexual intimacy”^3^,^4^. In addition, PE affects numerous aspects of a man's life, including sexual confidence, interpersonal relationships, and the sexual satisfaction of both partners^5^. The role of PE on the individual and the sexual relationship is very significant. Thus, it is important to treat patients with PE in order to improve quality of life. At present, treatment of PE includes mainly psychotherapy, drug therapy, and surgical treatment^6^.

Drug therapy is not only likely to be the most receptive approach for patients, but it is also the most commonly used method. Selective serotonin reuptake inhibitors (SSRIs) have become the most widely used medicine in the world^7^. Dapoxetine hydrochloride, belonging to the class of SSRIs, was the first drug originally approved for the on-demand treatment of men with PE by seven European countries in 2008^8^.

Unfortunately, the efficacy and safety of dapoxetine (30 mg and 60 mg on-demand) has never been comprehensively studied in men with PE. Most data derived from clinical studies in men with PE are available. The objective of this study was to not only evaluate the efficacy and safety of dapoxetine when used at either 30 mg or 60 mg as compared with the placebo as an oral on-demand treatment in men with PE in routine clinical practice by performing a meta-analytic synthesis of studies, but also to assess whether there are differences in efficacy and safety for PE treatment using either 30 mg or 60 mg dapoxetine.

## Results

**Figure 1:**
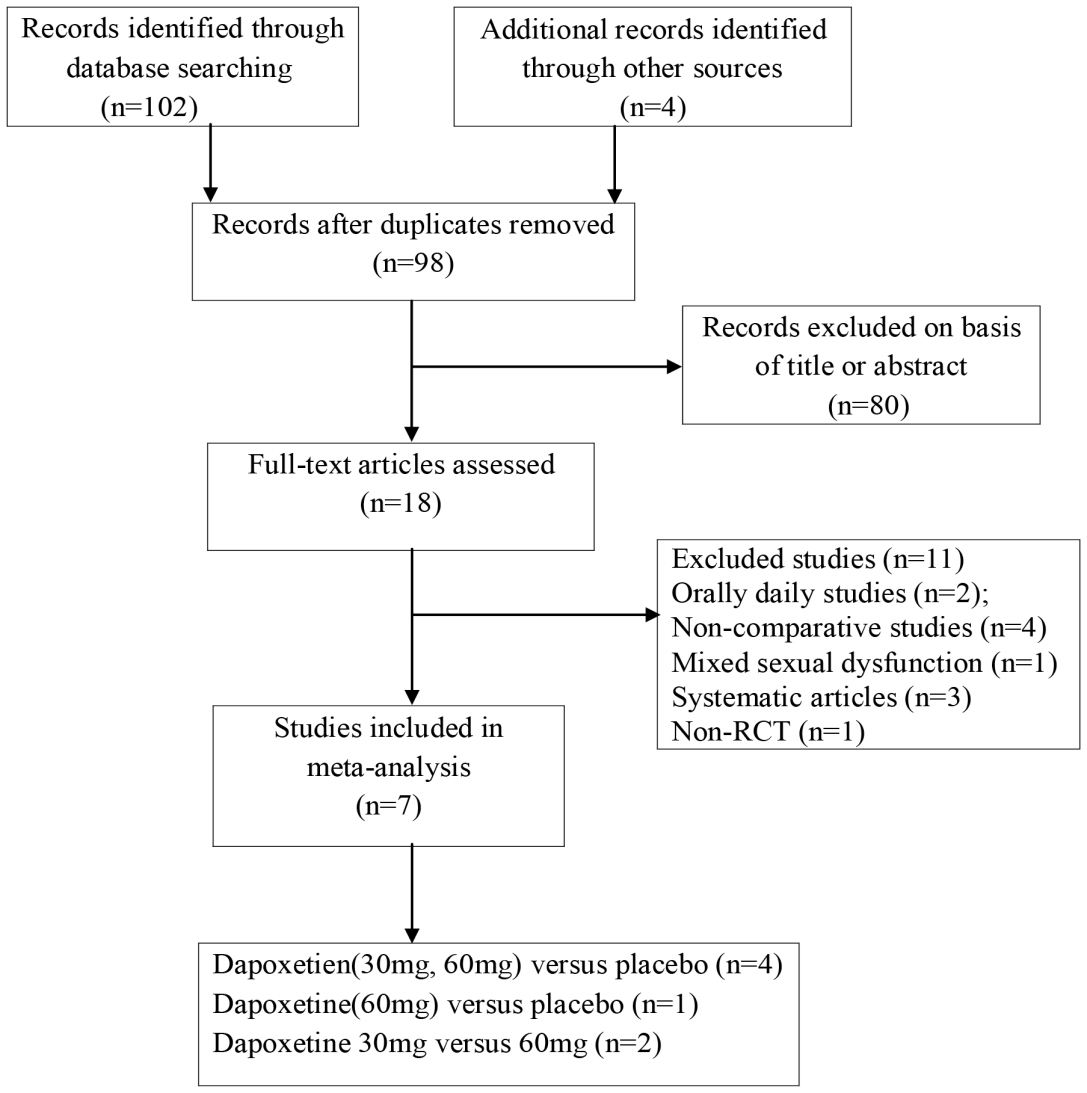
Flow diagram of evidence acquisition.

**Figure 2:**
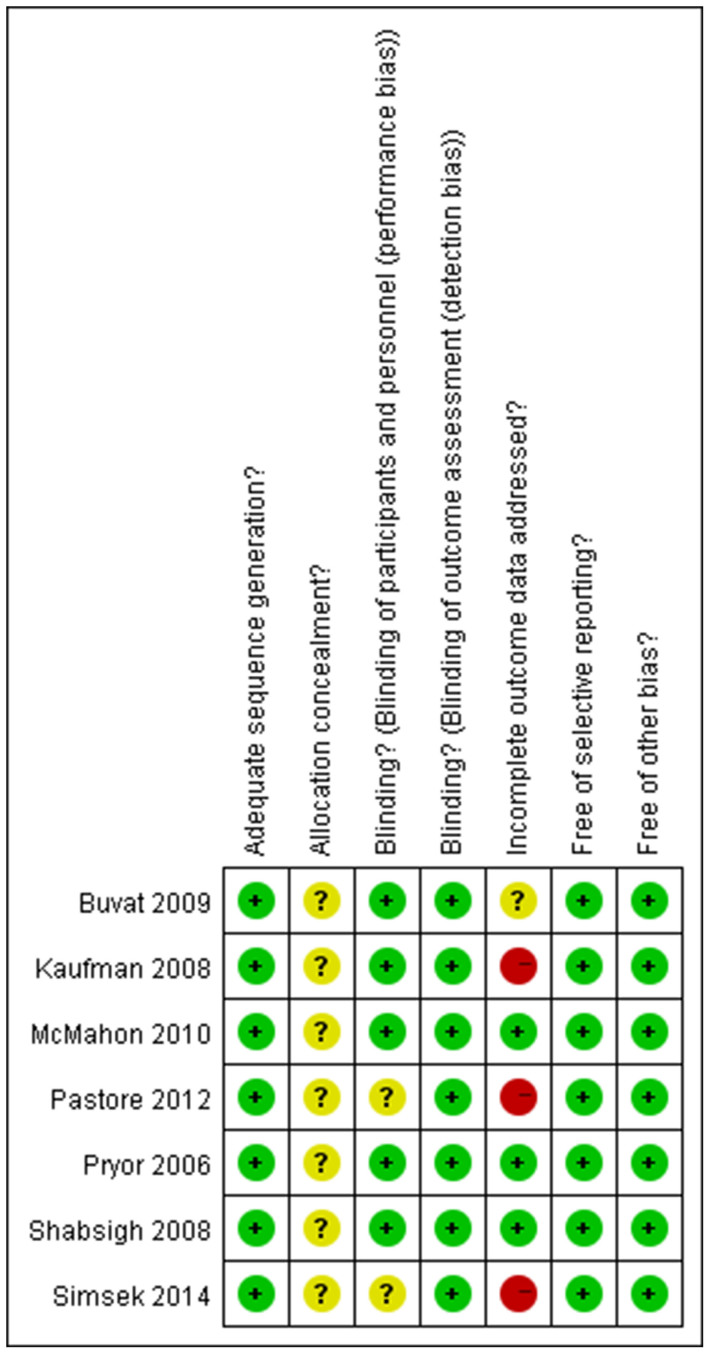
Risk of bias summary for each included study. + indicates low risk of bias, − indicates high risk of bias, and? indicates unclear risk of bias.

The electronic and manual searches of PubMed, EMBASE, and Cochrane Central Register of Controlled Trials (Cochrane Library) databases resulted in 106 references, of which 88 were clearly not relevant to our study. Of the remaining 18 references, 11 were excluded after reading the full text. After a quality validation, only seven studies^9^,^10^,^11^,^12^,^13^,^14^,^15^ were selected from the literature search. The flow chart of the evidence acquisition process is summarized in Figure 1, and the methodological quality of the included studies is reported in Figure 2.

Seven randomized controlled trials (RCTs) involving 8039 PE patients met the inclusion criteria. Of these, 2478, 2932, and 2629 patients received 30 mg dapoxetine, 60 mg dapoxetine, or the placebo, respectively. The study included patients aged 18 years or older and the patients were randomized for dapoxetine 30 mg or dapoxetine 60 mg or placebo administration on-demand (1–3 hours prior to anticipated sexual activity). The basic characteristics of the included studies are summarized in Table 1.

## Efficacy Assessments

### Intravaginal ejaculatory latency time (IELT)

**Figure 3:**
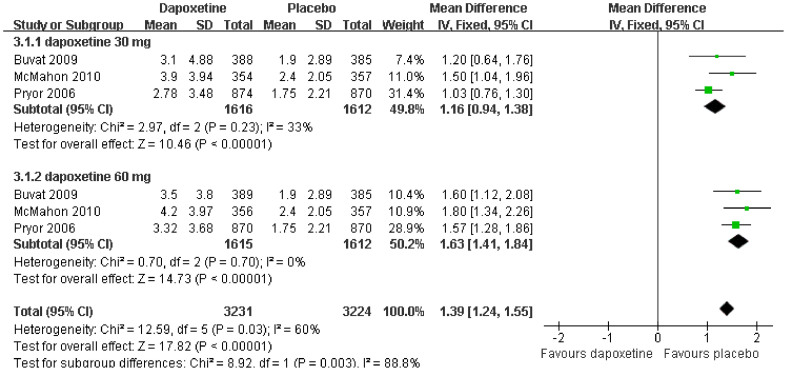
Forest plot of IELT between the dapoxetine (30 mg and 60 mg subgroup) and placebo group.

Three of the RCTs selected^9^,^12^,^13^ evaluating dapoxetine versus placebo for PE reported IELT as the primary outcome. Our pooled analysis showed that PE in patients in the dapoxetine group showed a significant improvement in IELT when compared to patients in the placebo group (mean difference [MD] = 1.39; 95% confidence interval [95% CI] = 1.24–1.55; P < 0.00001). Among these studies, we carried out a subgroup analysis based on PE patients treated with dapoxetine 30 mg and 60 mg on-demand oral administration. A statistically significant difference was found in the subgroup treated with 30 mg dapoxetine compared with the placebo-treated group (MD = 1.16; 95% CI = 0.94–1.38; P < 0.00001). The subgroup analysis of the dapoxetine group treated with 60 mg compared with placebo also reporting IELT also revealed a statistically significant difference in patient response (MD = 1.63; 95% CI = 1.41–1.84; P < 0.00001). (Figure 3).

**Figure 4:**
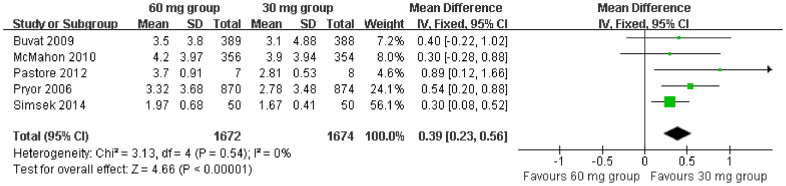
Forest plot of IELT between the 60 mg and 30 mg dapoxetine group.

In addition, five studies^9^,^12^,^13^,^14^,^15^ including a total of 3346 patients, we pooled to compare IELT; patients were divided into two groups treated with either 60 mg or 30 mg dapoxetine. In the fixed-effect model meta-analysis of the five studies, the pooled estimates were statistically significantly different between the dapoxetine 60 mg and 30 mg groups (MD = 0.39; 95% CI = 0.23–0.56; P < 0.00001; Figure 4). This pooled analysis indicated that the dapoxetine 60 mg group was associated with a markedly longer IELT than the dapoxetine 30 mg group.

### Patient-reported global impression of change (PGIC)

**Figure 5:**
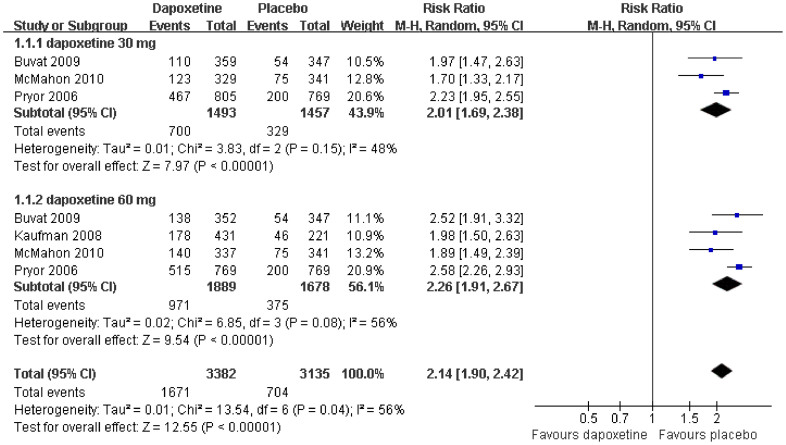
Forest plot of PGIC between the dapoxetine (30 mg and 60 mg subgroup) and placebo group.

Three studies^9^,^12^,^13^ including 2950 patients compared the PGIC in dapoxetine 30 mg and placebo subgroups, and four studies^9^,^10^,^12^,^13^ including 3567 patients compared PGIC in dapoxetine 60 mg and a placebo subgroup. Pooled analysis indicated that when compared to the placebo group, there was a significantly higher proportion of PGIC in the dapoxetine group. The overall risk ratio (RR) was 2.14 (95% CI = 1.90–2.42; P < 0.00001). The subgroup analysis indicated a statistically significant difference between the dapoxetine (for both 30 mg and 60 mg groups) and the placebo group in PGIC (RR = 2.01, 95% CI = 1.69–2.38, P < 0.00001; and RR = 2.26, 95% CI = 1.91–2.67, P < 0.00001, respectively; Figure 5).

**Figure 6:**
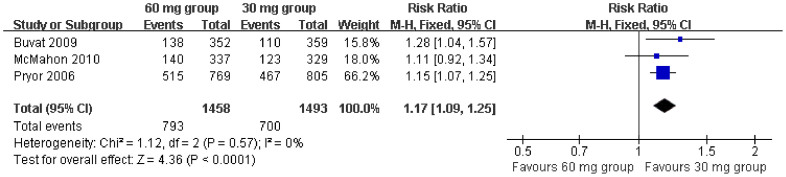
Forest plot of PGIC between the 60 mg and 30 mg dapoxetine group.

In addition, data from three^9^,^12^,^13^ studies reporting PGIC compared dapoxetine dosages (60 mg versus 30 mg). These count data were extracted to perform a forest plot analysis, which showed that the use of dapoxetine 60 mg was associated with a significantly greater improvement in PGIC than when dapoxetine 30 mg was used (RR = 1.17, 95% CI = 1.09–1.25; P < 0.00001, Figure 6).

## Safety Assessments

### Drug-related adverse effects (AEs)

**Figure 7:**
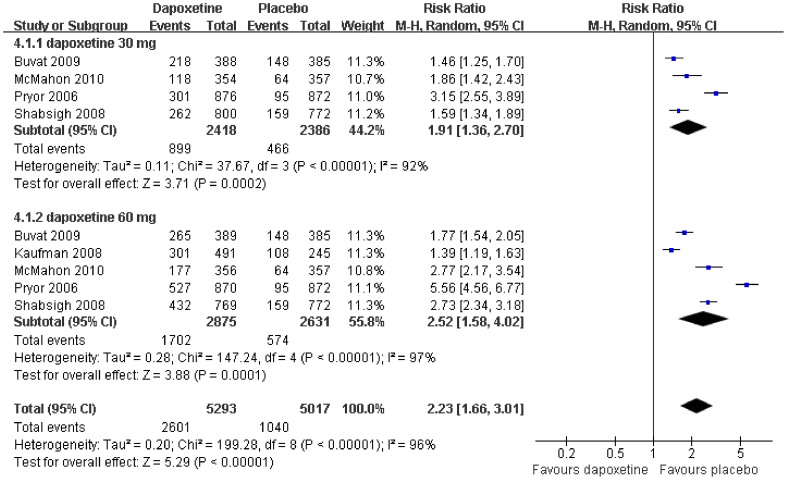
Forest plot of AEs between the dapoxetine (30 mg and 60 mg subgroup) and placebo group.

**Figure 8:**
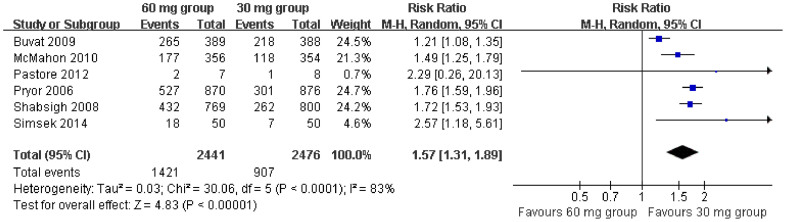
Forest plot of AEs between the 60 mg and 30 mg dapoxetine group.

Data from four^9^,^11^,^12^,^13^ and five^9^,^10^,^11^,^12^,^13^ studies reported AEs with sufficient data to generate a subgroup forest plot for dapoxetine 30 mg versus placebo and dapoxetine 60 mg versus placebo, respectively. Our pooled result of the meta-analysis showed that the number of AEs of patients in the dapoxetine (30 mg and 60 mg) group were significantly higher than those reported by patients in the placebo group (RR = 2.23; 95% CI = 1.66–3.01; P < 0.00001). The subgroup analysis indicated a statistically significant difference between treatment, with regards to 30 mg or 60 mg dapoxetine versus the placebo group in number of AEs reported (RR = 1.91, 95% CI = 1.36–2.70, P = 0.0002; and RR = 2.52, 95% CI = 1.58–4.02, P = 0.0001, respectively; Figure 7). Furthermore, AEs were assessed in six studies^9^,^11^,^12^,^13^,^14^,^15^ comparing two dapoxetine dosages (60 mg vs. 30 mg). This present plot demonstrated that a statistically significant difference existed between the 60 mg and 30 mg group in terms of the incidence of AEs (RR = 1.57; 95% CI = 1.31–1.89; P < 0.00001; Figure 8).

## Discussion

Dapoxetine hydrochloride was the first drug originally approved for the on-demand treatment of patients with PE in 2008. Since then, it has received marketing authorization in 59 countries worldwide^7^,^8^. In 2011, McMahon et al.^16^ published an integrated analysis of results from five phase 3 trials attempting to explore the efficacy and safety of dapoxetine for treatment of PE, and they proposed that dapoxetine significantly improved all aspects of PE and was generally well tolerated. In 2012, McCarty et al^17^ published a systematic descriptive review, and analyzed whether dapoxetine could be considered an efficacious and tolerable treatment for PE. However, no valid conclusions from these studies comparing 60 mg versus 30 mg dapoxetine could be obtained since these lacked strong statistical evidence.

In this meta-analysis, in order to obtain a reliable and scientifically sound comparison of dapoxetine (30 and 60 mg) versus placebo and dapoxetine 60 mg versus dapoxetine 30 mg as on-demand oral treatment for PE, a precise search strategy was performed to include all relevant RCTs. Therefore, studies of patients with erectile dysfunction^18^, non-RCTs^19^ and studies including patients with chronic, daily oral treatment with dapoxetine^20^,^21^ were excluded from the analysis of patients with PE. Ultimately, seven RCTs met the inclusion criteria for the present meta-analysis. To our knowledge, this is the most recent systematic review and meta-analysis comparing oral dapoxetine on-demand with placebo, and comparing dapoxetine dosages (60 mg versus 30 mg) for the treatment of patients with PE.

The present meta-analysis for IELT demonstrated that on-demand oral treatment with dapoxetine had an advantage over placebo despite the different dosages (30 mg and 60 mg) used. It demonstrated that on-demand oral dapoxetine was an effective treatment for PE. In addition, our meta-analysis comparing dapoxetine 60 mg with 30 mg on-demand orally proved that there was a statistically significant difference in IELT between the both groups; thus, 60 mg dapoxetine had a longer IELT than 30 mg on-demand for PE. The present findings are in agreement with the results of previous clinical trials that have reported significant improvement in the IELT with the use of dapoxetine^9^,^10^,^11^,^12^,^13^,^14^,^15^,^16^,^17^,^18^,^19^,^20^,^21^. Mean IELT was also significantly increased in all the studies not included in this meta-analysis. Mirone et al^22^ performed non-RCT trials in 2014 that compared dapoxetine 30–60 mg with alternative care/nondapoxetine, the results revealed that dapoxetine for treatment of PE had a longer mean IELT. The integrated analysis from five trials by McMahon et al^19^ also showed dapoxetine 30 and 60 mg on-demand significantly increased mean IELT compared with placebo (1.9 minutes for placebo, 3.1 and 3.6 minutes for dapoxetine 30 mg and 60 mg, respectively).

Our statistical results and subgroup analysis of PGIC demonstrated that dapoxetine was associated with a major improvement in PGIC. As shown in Figure 5, the overall RR was 2.14 (95% CI = 1.90–2.24) between dapoxetine and placebo, 2.01(95% CI = 1.69–2.38) between the dapoxetine 30 mg subgroup and placebo, and 2.26 (95% CI = 1.91–2.67) between the dapoxetine 60 mg subgroup and placebo, respectively. Additionally, the present meta-analysis also demonstrated significant improvement in PGIC with 60 mg over 30 mg dapoxetine on-demand alone. Pryor et al^9^ thought that the PGIC condition as a study endpoint is informative with respect to men's perception to minor detectable changes in IELT. In other words, there is a positive relationship between the participants' PGIC ratings and mean change in IELT values. The present findings agree with the results of previous clinical trials that reported a similar improvement in PGIC with dapoxetine versus placebo or comparing 60 mg versus 30 mg dapoxetine^9^,^10^,^11^,^12^,^13^,^16^,^17^,^18^,^19^,^20^,^21^.

This analysis revealed the incidence of total AEs was more frequent with dapoxetine than with placebo, and more common with dapoxetine 60 mg than 30 mg. However, from a review of all the studies reported in the literature, AEs of dapoxetine are generally tolerable. In the integrated analysis of the McMahon et al^16^ study, total AEs occurred in 35.1%, 47.0%, 60.3% subjects with placebo, dapoxetine 30 mg, and dapoxetine 60 mg on-demand orally, respectively. In Figure 7, the total number of AEs occurred in 49.1% (2601/5293) and 20.7% (1040/5017) of subjects treated with dapoxetine and placebo, respectively. In Figure 8, the total number of AEs occurring with dapoxetine 60 mg or 30 mg were 58.2% (1421/2441) and 36.6% (907/2476), respectively. The most frequently reported AEs were nausea, dizziness, headache, diarrhea, and insomnia^22^,^23^. Fortunately, the most common AEs with dapoxetine were mild or moderate in nature and were transient symptoms^9^,^10^,^11^,^12^,^13^,^15^,^16^,^17^,^18^,^19^,^20^,^21^,^24^,^25^. The present findings agree with the results of previous clinical trials that reported similar low incidence rates of side effects.

Dapoxetine is a short-acting SSRI and doses of 30 mg and 60 mg have been evaluated through our meta-analysis. Peak plasma concentrations of dapoxetine were observed within 1.01–1.27 hours after oral administration^24^. The elimination half-life time is 1.3–1.4 hours and there appears to be very little accumulation^24^. Dapoxetine undergoes rapid absorption, elimination, and dose-dependent pharmacokinetics, which are unaffected by multiple dosing. The unique pharmacokinetic characteristics might be the reason why dapoxetine is the on-demand treatment of choice for PE. Safety and efficacy data have demonstrated that dapoxetine use produces acceptable improvement in the IELT and PGIC with on-demand use. Thus, we also believe it is probably better suited as an on-demand treatment option for PE^26^.

Our systematic review and meta-analysis has many drawbacks, the primary one being that this was a heterogeneous trial. In an attempt to reduce the high heterogeneity, we carried out a subgroup analysis among studies involving the drug dosage used (30 mg versus 60 mg groups). The heterogeneity was significantly decreased when comparing dapoxetine with placebo in the IELT by subgroup analysis. However, this heterogeneity of data still exists in the PGIC and AEs analyses, which we were unable to improve. We believe this heterogeneity might have resulted in the evaluation of the PGIC value using a 7-point scale (from −3 = much worse to 3 = much better) and AEs that were assessed using a subjective evaluation by individual patients. Additional factors might have been potentially amplified heterogeneity; these include differences in treatment duration, individual differences, and mental or physical conditions. Secondly, some of the included studies did not report the outcome measures; hence, the statistical results might be influenced by the statistical parameters used for calculations. Additionally, nearly all trials included in this study lacked a clear description of the allocation concealment, but all trials included in this meta-analysis were RCTs, the methods were designed well. Thus, the data from the studies included in our meta-analysis were reliable.

In summary, our meta-analysis has shown that either 30 mg or 60 mg dapoxetine on-demand orally was associated with a significantly greater increase in mean IELT and PGIC compared placebo. Additionally, 60 mg had a better efficacy than 30 mg dapoxetine on-demand orally. However, the meta-analysis also demonstrated that those treated with dapoxetine (especially 60 mg on-demand orally) reported more AEs than placebo or the dapoxetine 30 mg group. Nonetheless, the most commonly reported AEs were mild and tolerated.

## Methods

### Search strategy

We searched the following databases up to and including June 2014: MEDLINE by PubMed, EMBASE, Cochrane Central Register of Controlled Trials (Cochrane Library). We did not restrict our search to articles published in English, and the following search terms were used in conjunction with: dapoxetine, SSRIs, and premature ejaculation, sexual dysfunction. We also searched the relevant references of all studies included in the analysis. All retrieval literatures were independently performed by Cao D and HY.

### Study selection

Included in the study were all published or unpublished RCTs evaluating dapoxetine interventions for PE. Studies comparing dapoxetine intervention versus placebo or another drug intervention were eligible for this review. All relevant studies were included in this study if they met the following criteria: (1) all patients were older than 18 years; (2) patients were diagnosed with PE; (3) patients were treated with oral dapoxetine on-demand (1–3 hours before sexual activity); (4) data were available for at least one of the predefined outcome measurements. Studies were excluded if (1) patients were diagnosed with mixed sexual dysfunction such as erectile dysfunction plus PE; (2) patients were treated with a fixed-dose orally daily; (3) the data referred to data from an animal study; or (4) studies reported data from non-RCTs or quasi-RCTs.

### Data extraction

The following variables from each study were recorded independently by two reviewers and cross-checked: first author name, publication year, research design type, total number of patients enrolled, patient age, intervention method, outcome measures. In addition, the following primary outcome was extracted: IELT, defined as the time from the start of vaginal insertion to the start of intravaginal ejaculation and measured using the stopwatch. The secondary outcomes were as follows: PGIC, also called the clinical global impression of change (CGIC or CGI) in some studies, defined as a validated tool used to measure overall perceived change in patients with better and much better results after treatment (i.e., “Compared to the start of the study, would you describe your PE problem as much worse, worse, slightly worse, no change, slightly better, better, or much better?^27^”), and AEs, defined as potential symptoms related to dapoxetine discontinuation syndrome such as nausea, diarrhea, insomnia, headache, dizziness, erectile dysfunction, fatigue.

### Quality assessment

The methodological quality of the included studies was measured independently by two reviewers using the Cochrane Handbook for Systematic Reviews of Interventions^28^. The main evaluation items included: (1) random sequence generation (selection bias), (2) allocation concealment (selection bias), (3) blinding of participants (performance bias), (4) blinding of outcome assessment (detection bias), (5) incomplete outcome data (attrition bias), (6) selective reporting (reporting bias) and (7) other bias. These criteria for a judgment of low, high, or unclear risk of bias for each item were used to describe the bias. A “Yes,” “No,” or “Unclear” assessment expressed as low risk of bias, high risk of bias, or uncertain risk of bias, respectively. Disagreements were discussed and resolved by using a third person-evaluation. These assessments were reported for each individual study in the “risk of bias in included studies” in Figure 2.

### Statistical analysis

All statistical analyses were conducted using Review Manager, version 5.1.0 (Cochrane Collaboration, Oxford, UK). Statistical analysis of dichotomous variables (PGIC and AEs) were performed using the RR as the summary analysis, while continuous variable (IELT) was analyzed using the MD; accompanying 95% CIs and P-values were reported. For all statistical results, P < 0.05 was considered statistically significant. The Mantel-Haenszel χ^2^ test and *I^2^* statistic for heterogeneity were conducted. *I^2^* values of <50% were defined as acceptable; those >50% indicated high levels of heterogeneity. When there was a lack of heterogeneity, a fixed-effects models was used, otherwise random-effects model was applied for the meta-analysis.

## Author Contributions

W.Q. contributed to the conception and design of the study, C.D. and L.L. wrote the main manuscript text, and H.Y. and C.D. prepared figures 1–8. All authors reviewed the manuscript. C.D. and L.L. are contributed equally to this work.

## Figures and Tables

**Table 1:** Basic features and quality assessments of the included studies

Study, year	Designs	Invention	Patients (n)	Age (years)	TD (week)	Outcomes measures
Pryor,^2006^	RCT	D 30, 60 mg vs P	878, 870 vs 870	40.3(9.10), 40.9(9.09) vs 40.3(9.55)	12	IELT, PGIC, AEs
Kaufman,^2008^	RCT	D 60 mg vs P	491 vs 245	40.9(9.71) vs 41.8(9.80)	9	PGIC, AEs
Shabsigh,^2008^	RCT	D 30, 60 mg vs P	800, 769 vs 772	≥18, ≥18 vs ≥18	12	IELT, PGIC, AEs
Buvat,^2009^	RCT	D 30, 60 mg vs P	388, 389 vs 385	39.6(9.53), 40.5 (9.62) vs 40.1(9.98)	24	IELT, PGIC, AEs
McMahon,^2010^	RCT	D 30, 60 mg vs P	354, 356 vs 357	41.2(10.74), 41.0(10.78) vs 40.6(9.71)	12	IELT, PGIC, AEs
Pastore,^2012^	RCT	D 30 vs 60 mg	8 vs 7	31(23-51) vs 31(23-51)	12	IELT
Simsek,^2014^	RCT	D 30 vs 60 mg	50 vs 50	33.5 (3.45) vs 32.4 (2.90)	4	IELT, AEs

RCT = randomisd controlled trial; D = dapoxetine, orally, on demand 1–3 hours sexual intercourse; P = placebo, orally, on demand 1–3 hours sexual intercourse; vs = versus; TD = treat duration; IELT = intravaginal ejaculatory latency time; PGIC = patient-reported global impression of change; AEs = drug-related adverse effects.
